# Ferritinophagy: a possible new iron-related metabolic target in canine osteoblastic osteosarcoma

**DOI:** 10.3389/fvets.2025.1546872

**Published:** 2025-03-24

**Authors:** Karen Power, Rebecca Leandri, Giorgia Federico, Gionata De Vico, Leonardo Leonardi

**Affiliations:** ^1^Department of Biology, University of Naples Federico II, Naples, Italy; ^2^Department of Molecular Medicine and Medical Biotechnologies, University of Naples Federico II, Naples, Italy; ^3^Department of Veterinary Medicine, University of Perugia, Perugia, Italy

**Keywords:** bone cancer, canine osteosarcoma, immunohistochemistry, iron metabolism, therapy

## Abstract

Canine osteosarcomas (COS) are the most common bone tumors in dogs, characterized by high metastatic rates, poor prognosis, and poor responsiveness to routine therapies, which highlights the need for new treatment targets. In this context, the metabolism of neoplastic cells represents an increasingly studied element, as cancer cells depend on particular metabolic pathways that are also elements of vulnerability. Among these, tumor cells (TCs) show higher iron requirements to sustain proliferation (so-called iron addiction), which are achieved by increasing iron uptake and/or by activating ferritinophagy, a process mediated by the Nuclear receptor Co-Activator 4 (NCOA4) leading to iron mobilization from ferritin (Ft) deposits. Previous studies have shown that COS cells overexpress Transferrin Receptor 1 (TfR1) to increase iron uptake. In this study we evaluated the immunohistochemical expression of ferritinophagy-related proteins, namely Ferritin Heavy chain (FTH1) and NCOA4, and proliferating cell nuclear antigen (PCNA) in canine normal bone and canine osteoblastic osteosarcoma (COOS) samples. Normal samples revealed negative/weak immunoreactivity for FTH1, NCOA4 and PCNA in <10% of osteocytes. In COOS samples the majority of neoplastic cells showed immunoreactivity to FTH1, NCOA4 and PCNA. Our data suggest that the activation of ferritinophagy by COOS cells responds to the need for feed their “iron addiction.” These data, though preliminary, further suggest that targeting iron metabolism represents a new potential strategy worthy of further study to be transferred into clinical practice.

## 1 Introduction

The study of metabolic alterations of neoplastic cells is currently a hot topic, as cancer cells can become addicted to specific metabolic pathways also representing metabolic vulnerabilities against which novel drugs that target them can be developed (1). Among these, the so-called “iron addiction” is one of the most relevant metabolic alterations of neoplastic cells (2). Cancer cells show higher iron requirements than normal cells to sustain proliferation (3) and tissue invasion (4) and tend to satisfy this need by over-expressing a series of proteins involved both in the iron uptake from the bloodstream (5, 6) and in its mobilization from intracellular reserves by so-called “ferritinophagy,” a selective form of autophagy that specifically targets intracellular (Ft) for lysosomal degradation (7, 8). Key molecules in iron metabolism are: (1) TfR1, which uptakes and internalizes iron by binding transferrin (Tf)-Fe3+ complex, which is followed by Fe3+ reduction to Fe2+ by ferrireductases in the cytosol (9); (2) the Ft, which represents the storage site of iron in the cytosol, and which also contributes to the physiological release of iron from reserves to form the cytoplasmic labile iron pool (cLIP) (10–12); and (3) the NCOA4, a selective cargo protein which binds to a conserved C-terminal domain of FTH1 and to autophagy-related proteins to deliver FT to autophagosomes and trigger ferritinophagy (13, 14). Previous studies in human pathology have reported impairment of iron metabolism in different cancers (15–21). This appears to be particularly true in human osteosarcomas (22, 23), the most common primary malignant bone tumor affecting children and adolescents (24, 25). Unfortunately, in veterinary medicine iron metabolism and its alterations connected to cancer are still poorly studied (26–31). The early results presented in a previous study (26) highlighted the relevance of TfR-1 expression in canine osteosarcomas (COS), suggesting therapies involving both TfR-1 and other molecules related to iron metabolisms in dogs with osteosarcoma should be developed, also considering the potential clinical impact for humans. COS represent a well-known preclinical model for human osteosarcoma, particularly for those developing in young people as they share molecular and morphological aspects, as well as prognosis and treatment options (32). COS represent the most frequent primary malignant bone neoplasms of mesenchymal origin in dogs (33, 34), exhibiting local aggressiveness, high metastatic behavior and high mortality rates (35–38). COS originate mainly from appendicular skeleton, with the most frequent localization occurring at the metaphyseal level, while only 20–25% of tumors originate from the axial bone (34). Histological classification of bone tumors of domestic animals describes the presence of six different histotypes, namely: poorly differentiated, osteoblastic (productive and non-productive), chondroblastic, fibroblastic, telangiectatic, giant cell type, with the osteoblastic type being the most frequent (33, 39). To date, therapy is based on surgery (conservative or not) coupled to chemotherapy and radiotherapy, however life expectancy remains low (40–42) and resistance to typical antineoplastic drugs is building up (43–45). Therefore, the need for new targets, new antineoplastic drugs and/or adjuvant antineoplastic compounds for COS is rising. In this context, we recently validated and studied the expression of the NCOA4 and FTH1 in some canine normal and neoplastic tissues (46). In this report, we provide additional evidence for the relevance of iron metabolism alterations in canine osteoblastic osterosacomas (COOS), highlighting the role of ferritinophagy-related molecules NCOA4 and FTH1, thus suggesting that the mechanisms of ferritinophagy could represent a further potential pathway to be targeted to selectively destroy this type of cancer cells.

## 2 Materials and methods

### 2.1 Tissue samples

Three normal bone samples (N1-N3) and 20 COOS samples (COOS1-COOS20) were retrieved from the archives of the Department of Veterinary Medicine – University of Perugia. Ethics committee’s approval and animal testing request were waived since all animal tissue samples examined in this study were retrieved from archives. Samples had been previously decalcified and processed by routing histological techniques, paraffin-embedded and stained with hematoxylin and eosin (H&E). All samples had been observed by light microscopy for morphological classification of histological subtypes according to the World Health Organization’s histologic classification of tumors of domestic (33).

### 2.2 Immunohistochemistry

For each paraffin-embedded sample 3 μm sections were processed for immunohistochemistry (IHC) as previously described (47) to evaluate expression of proteins involved in ferritinophagy (FTH1, NCOA4), and PCNA to assess proliferation (46). Antibody specification and dilutions are reported in Table 1. Sections were counterstained with hematoxylin, and immunolabeling was revealed with diaminobenzidine-tetrahydrochloride (DAB).

**Table 1:** Antibodies used in immunohistochemical analysis.

Antibody	Manufacturer/clone	Host species	Dilution	
FTH1	Antibodies/Polyclonal	Rabbit	1:100	Leandri et al. (46)
NCOA4	Abcam ab62495/439CT10.1.2	Mouse	1:100	Leandri et al. (46)
PCNA	Abcam ab18197/PC10	Mouse	1:400	Ersoy et Ozem (68)

### 2.3 Scoring of Immunoreactivity

To evaluate the expression of FTH1, NCOA4 and PCNA a semiquantitative score was applied by analyzing the number of positively labelled cells in 1,000 cells in 10 fields at 400x magnification (40x objective 10x ocular) for each specimen by two independent observers (Leonardo Leornardi and Gionata De Vico) under blinded conditions (48). Results were expressed as percentage.

## 3 Results

### 3.1 Histopathology results

**Figure 1:**
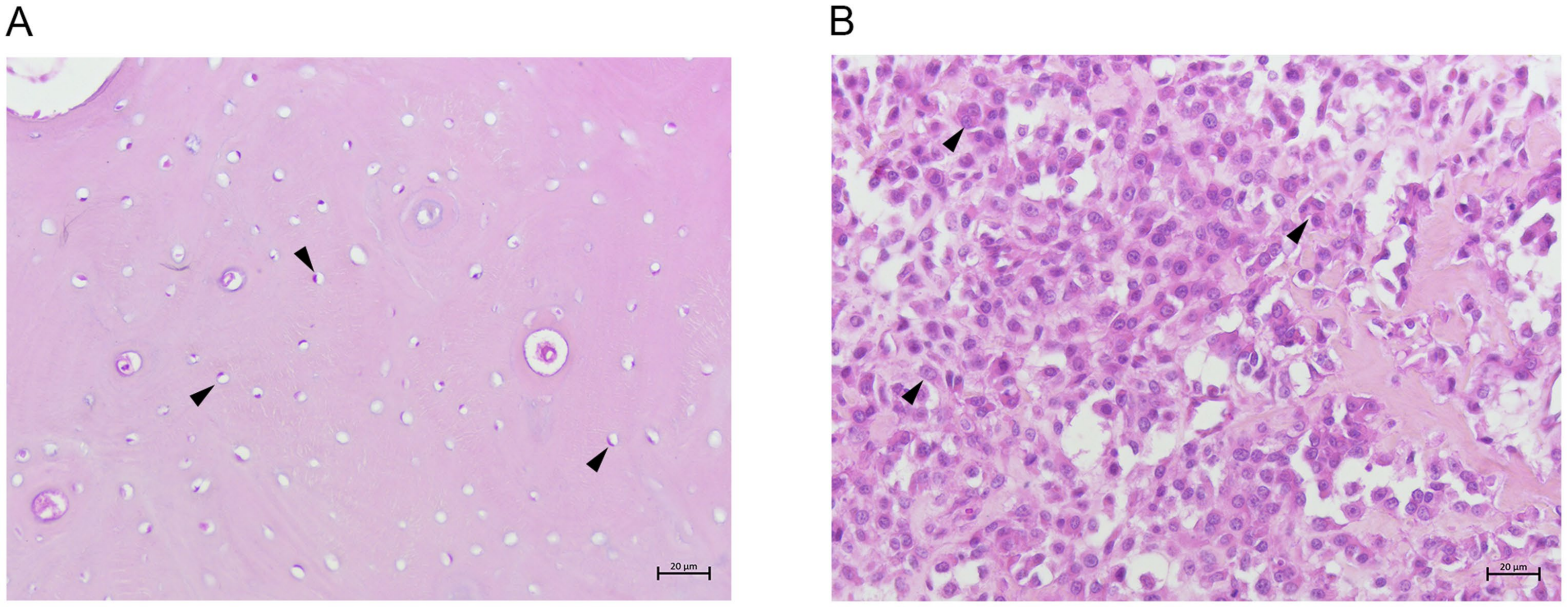
**(A)** Canine normal bone tissue showing osteocytes (arrow heads) and abundant bone matrix. H&E 20x. **(B)** Canine productive osteoblastic osteosarcoma showing many polyhedral cells (arrow heads) and osseus matrix. H&E 20x.

Breeds, sex, age, tumor localization and histologic classification are summarized in Supplementary Table S1. Normal tissue samples (N1-N3) were characterized by abundant bone matrix in which elliptical osteocytes, showing mildly basophilic cytoplasm and oval nucleus, were immersed (Figure 1A). All COS samples (COOS1-COOS20) were characterized by polyhedral cells with eccentric nuclei and basophilic cytoplasm. Nuclei appeared pleomorphic, presenting hyperchromatic chromatin, and bizarre and atypical mitosis were observed. Osseus matrix was present in moderate to high amounts, often in the pattern of dense sheets (Figure 1B). Considered the histopathological features observed in the COS samples, they were classified as productive COOS.

### 3.2 Immunohistochemistry results

**Figure 2:**
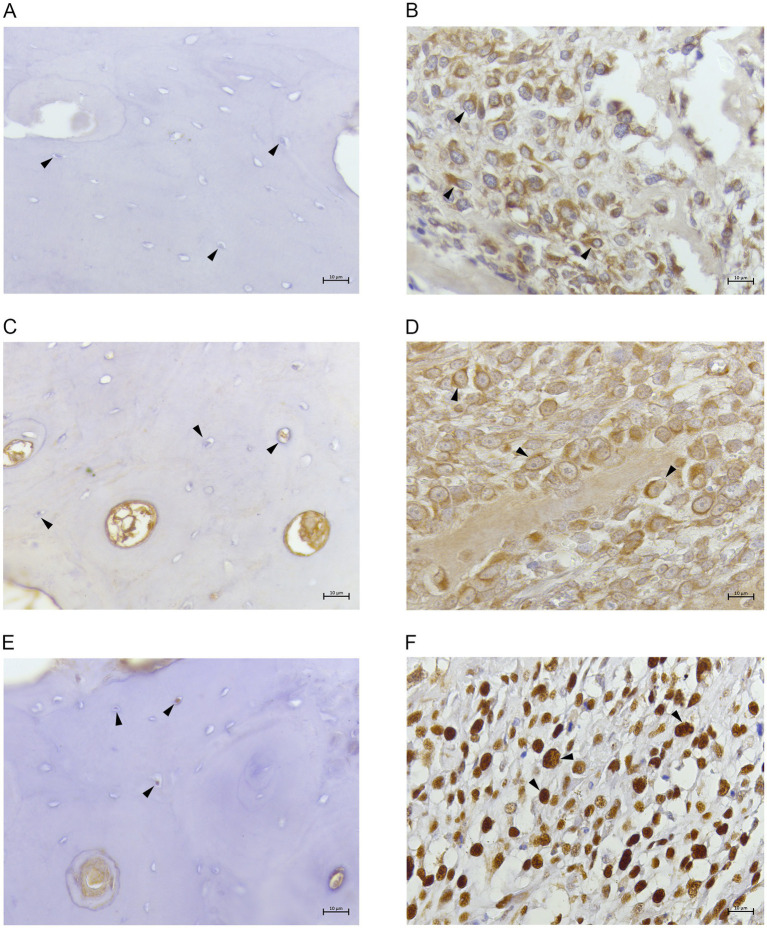
**(A)** Canine normal bone tissue. FTH1. Osteocytes showing no immunolabeling. 40x; **(B)** canine productive osteoblastic osteosarcoma. FTH1. Tumoral cells revealed cytoplasmic immunostaining (arrow heads). 40x; **(C)** canine normal bone tissue. NCOA4. Osteocytes showing no/weak immunolabeling. 40x; **(D)** canine productive osteoblastic osteosarcoma. NCOA4. Tumoral cells revealed cytoplasmic/perinuclear immunostaining (arrow heads). 40x; **(E)** canine normal bone tissue. PCNA. Few osteocytes showing weak nuclear immunolabeling. 40x. **(F)** canine productive osteoblastic osteosarcoma. PCNA. Tumoral cells showing strong nuclear immunolabeling (arrow heads). 40x.

Normal bone samples presented less than 10% of cells positive for all the three tested antibodies (Figures 2A,C,E). On the contrary, in COOS samples 85–95% of neoplastic cells showed a strong cytoplasmic immunostaining for FTH1 (Figure 2B) and NCOA4. (Figure 2D). Moreover, 70–80% of neoplastic cells were strongly labelled at the nuclear level by anti-PCNA (Figure 2F).

## 4 Discussion

Canine osteosarcomas (COS) are aggressive malignancies of the bone, for which the prognosis of patients still remains relatively poor and survival rates have not significantly improved during the recent decades. COS share biological and clinical similarities with the human counterpart, where a growing research tendency is focusing on the role of iron and its metabolism in both tumor progression and tumor suppression (2, 3, 20). Given the similarities between the two species, we investigated the expression in COOS of key proteins involved in iron metabolism to possibly identify new therapeutical targets for both dogs and possibly humans. Our results show an increased expression of all analyzed proteins in COOS samples compared to normal samples. Previous data on the overexpression of TfR1 in COS (26), supported the idea that iron uptake plays a decisive role in supporting the growth of COOS neoplastic cells and could represent a new therapeutic target. Our study emphasizes for the first time in COOS the role of NCOA4 and FTH1, key molecules involved in ferritinophagy regulation (49). Interestingly, in our study cancer samples showed higher immunoreactivity in neoplastic cells compared to normal ones, in accordance to literature (50, 51). In the classical ferritinophagy pathway NCOA4 interacts with ferritin-heavy chain (FTH1), transferring autophagosomes to lysosomes to degrade FT and release free iron thus increasing cLIP. Physiologically, NCOA4 combined with iron is continuously degraded by ubiquitin-proteasome system or directly by lysosomes (52), explaining why in our study NCOA4 was usually poorly highlighted in normal cells by immunohistochemistry. On the contrary, an intriguing result of our investigation is the strong immunohistochemical detection of NCOA4 coupled with the one of FTH1 in COOS cells, which testify for a deep dysregulation of iron metabolism and in particular of the ferritinophagy pathway. In our case, in fact, it could be hypothesized that the COOS cells are so highly dependent on the availability of iron for their growth and survival (iron addiction), to simultaneously activate different pathways that allow them to maintain high levels of iron in the cytosol, namely iron upload, storage and mobilization from storage. High iron loads and ferritinophagy have also been closely correlated with ferroptosis, a form of iron dependent non-apoptotic programmed cell death linked to oxidation of membrane lipid (53). It is to be believed that COOS cells have developed mechanisms to evade these forms of cell death as already described in other tumor types (54, 55, 69). As a matter of fact, in our cases there was no evidence of characteristic morphological feature of ferroptosis in COS cells, namely cell membrane rupture, cytoplasmic swelling, and moderate chromatin condensation (56). Escaping ferroptotic mechanisms provides further vulnerable possible targets for ferroptosis-based therapy (70). Previous studies in human oncology have described the possibility of using synthetical or natural compounds to target iron metabolism (57–60) and enhance ferroptosis. Artemisin, the main bioactive component of *Artemisia annua* L, has been proven to activate apoptosis, ferroptosis and induce cancer cell death by producing ROS in human osteosarcoma (61, 62) and also in COS cell lines (63). More recently, two studies by Isani et al. (64) and Colurciello et al. (65) showed that COS cells treated with artemisin showed higher mortality rates and lower iron concentrations compared to untreated ones, probably due to ferroptosis. Furthermore, targeting ferritinophagy pathway can also represent mechanisms for some common anticancer drugs. As examples, low-dose cisplatin combined with ursolic acid inhibits cancer cell growth by activating autophagic degradation of Ft and overloading intracellular iron ions (66). The combination of artesunate and the hepatocellular carcinoma first-line drug sorafenib induces ferritinophagy in hepatocellular carcinoma cells and improve the efficacy of single anticancer drugs (67). The results of our study provide relevant, thought preliminary data on the alteration of the iron-metabolic pathway in COOS. Notably, they suggest an increased uptake of iron (26), release of iron from ferritin-storage coupled to a continuous replacement of the used Ft storage. COS appear as favorable candidates for the use of antineoplastic drugs targeting iron metabolism, ferroptosis and ferritinophagy. Ideally, therapies should on one hand enhance cLIP by increasing NCOA4-induced ferritinophagy and on the other hand use TfR1 as a tool to selectively deliver compounds to tumoral cells and reduce undesired effects on healthy cells. Further studies will help deepen the knowledge about alterations in iron metabolism in COOS. Of particular interest would be correlating the overexpression of these molecules with patient follow-up data to assess their potential prognostic implications, and using 2D cell models hopefully opening the way to possible *in vivo* studies to be transferred into clinical practice.

## Data Availability

The original contributions presented in the study are included in the article/Supplementary material, further inquiries can be directed to the corresponding author.
