# Supplementary material for: Ferritinophagy: a possible new iron-related metabolic target in canine osteoblastic osteosarcoma
Source: Front Vet Sci. 2025 Mar 24;12:1546872. doi: 10.3389/fvets.2025.1546872 (PMC11973301; doi:10.3389/fvets.2025.1546872)
Supplement: Supplementary file 1 [file Table_1.DOCX]

Supplementary Material

# Supplementary Table 1. Breed, age, sex, localization and canine osteoblastic osteosarcoma (COOS) type analyzed.

| **Sample** | **Breed** | **Age (ys)** | **Sex** | **Localization** | **Osteoblastic COS type** |
| --- | --- | --- | --- | --- | --- |
| N1 | Mixed breed | 7 | M | tibia | - |
| N2 | German Shepherd | 9 | F | tibia | - |
| N3 | Boxer | 9 | F | radius | - |
| COOS1 | Mixed breed | 11 | M | humerus | Productive |
| COOS2 | German Shepherd | 7 | F | tibia | Productive |
| COOS3 | Mixed breed | 12 | M | radius | Productive |
| COOS4 | Mixed breed | 10 | M | radius | Productive |
| COOS5 | Golden retriever | 12 | F | femur | Productive |
| COOS6 | Boxer | 11 | F | humerus | Productive |
| COOS7 | Saint Bernard | 8 | M | tibia | Productive |
| COOS8 | Rottweiler | 11 | F | radius | Productive |
| COOS9 | German Shepherd | 8 | F | femur | Productive |
| COOS10 | German Shepherd | 10 | M | tibia | Productive |
| COOS11 | Mixed breed | 7 | F | humerus | Productive |
| COOS12 | Rottweiler | 13 | F | femur | Productive |
| COOS13 | Terranova | 7 | M | tibia | Productive |
| COOS14 | Dobermann | 8 | M | radius | Productive |
| COOS15 | Terranova | 9 | F | humerus | Productive |
| COOS16 | Boxer | 9 | F | tibia | Productive |
| COOS17 | Mixed breed | 8 | F | tibia | Productive |
| COOS18 | German Shepherd | 7 | M | femur | Productive |
| COOS19 | Mixed breed | 9 | M | radius | Productive |
| COOS20 | German Shepherd | 10 | F | radius | Productive |

N1-N3: canine normal bone samples; COOS1-COOS20: canine osteoblastic osteosarcoma (COOS) samples; F: female; M: male.
